# Comprehensive Robustness Evaluation of Proton and Carbon-Ion Plans in Thoracic Cancer Treatment

**DOI:** 10.1016/j.ijpt.2025.101195

**Published:** 2025-06-24

**Authors:** Yinxiangzi Sheng, Lennart Volz, Jingfang Mao, Jian Chen, Timo Steinsberger, Weiwei Wang, Jiayao Sun, RongCheng Han, Marco Durante, Christian Graeff

**Affiliations:** 1 Biophysics, GSI Helmholtz Center for Heavy Ion Research GmbH, Darmstadt, Germany; 2 Department of Medical Physics, Shanghai Proton and Heavy Ion Center, Fudan University Cancer Hospital, Shanghai 201321, China; 3 School of Sensing Science and Engineering, Shanghai Jiao Tong University, Shanghai, China; 4 Department of Radiation Oncology, Shanghai Proton and Heavy Ion Center, Shanghai, China; 5 Shanghai Key Laboratory of Radiation Oncology (20dz2261000), Shanghai, China; 6 Shanghai Engineering Research Center of Proton and Heavy Ion Radiation Therapy, Shanghai China; 7 Institute of Condensed Matter Physics, Technical University of Darmstadt, Darmstadt, Germany; 8 Institute of Electrical Engineering and Information Technology, Technical University of Darmstadt, Darmstadt, Germany

**Keywords:** Robustness evaluation, Proton therapy, Carbon therapy, Motion mitigation, Pencil beam scanning

## Abstract

**Purpose:**

To evaluate the impact of setup errors, range uncertainty, and respiratory motion on dose distributions for clinically delivered pencil beam scanning proton and carbon-ion plans.

**Materials and Methods:**

A retrospective analysis was conducted on 23 lung cancer and trachea adenoid cystic carcinoma patients who received treatment at our center. Plans were generated using Syngo with planning target volume-based optimization. Dose reconstruction was performed using TReatment planning for Particles 4D. The plans robustness evaluation was performed using two methods: a worst scenarios conventional evaluation (WSCE) with 21 scenarios and a worst scenarios statistical evaluation (WSSE) with 100 randomly sampled scenarios. On top of the 3D evaluation considering setup error and range error, a 4D evaluation was performed considering motion-induced error.

**Results:**

The overall target dose ΔD95% was −2.37% ± 1.55% (mean ± standard deviation [SD]) of the prescribed dose (PD) and −2.62% ± 2.08% for 3DWSSE and 4DWSSE, respectively. The WSCE method often underestimated the dose by approximately 5% for ΔD95%. The induced uncertainties had limited impact on mean doses for Lungs-iGTV and heart. However, a ΔD1cc greater than 5% of PD was observed for the esophagus and trachea.

**Conclusion:**

Conventional robustness evaluation showed significantly reduced target coverage, yet it considers highly improbable worst-case scenarios. Comprehensive WSSE enables the identification of critical patients without compromising plan quality by avoiding overestimation and compensating for unrealistic error scenarios.

## Highlights

• Statistical robustness evaluation of clinically delivered pencil beam scanning proton/carbon plans.
• The worst scenarios statistical evaluation approach demonstrated potential dose variations in 90% of patients.
• Conventional worst scenarios often underestimate the target dose by 5%.
• Observed −18.2% ΔV95%, emphasizing the importance of proper robustness evaluation.

## Introduction

There has been a growing interest in utilizing proton and carbon-ion pencil beam scanning (PBS) for treating moving targets, particularly for patients with locally advanced diseases or those unsuitable for surgical intervention.^1^, ^2^, ^3^, ^4^, ^5^ However, PBS exhibits higher sensitivity to both inter-fractional and intra-fractional uncertainties, particularly in the case of anatomical motion. Interplay effects arise due to the interaction between the scanning pattern and the target motion.^6^, ^7^, ^8^, ^9^ Considering the significant impact of plan robustness on the accuracy and effectiveness of proton and carbon-ion therapy and its direct influence on clinical outcomes, evaluating the robustness of PBS plans becomes crucial.^10^

Numerous studies in the literature have reported various techniques for achieving robust planning and/or evaluating the robustness of proton and carbon-ion PBS plans.^11^, ^12^, ^13^, ^14^, ^15^ Meanwhile, substantial efforts have been dedicated to creating conformal plans in particle radiotherapy.^16^ However, clinical scenarios frequently necessitate a balance between plan quality/conformity and robustness.^17^ This compromise stems from balancing the plan's ability to achieve the desired dose distribution while accounting for setup uncertainties and patient-specific variations.

Despite the advancements in robust optimization techniques, many particle therapy centers—particularly carbon-ion centers—lack clinically available treatment planning system (TPS) that integrate 4D or robust optimization tools. As a result, retrospective evaluations of the robustness of clinically delivered plans are essential for identifying potential vulnerabilities and for optimizing future treatment approaches.

In this context, this study presents a retrospective analysis of the treatment plans of 23 thoracic cancer patients who received proton and/or carbon-ion PBS treatment with gated motion mitigation at the Shanghai Proton and Heavy Ion Center (SPHIC). The evaluation was conducted in the research TPS TReatment planning for Particles 4D (TRiP4D).^18^, ^19^ The study aims to evaluate the robustness of the clinically delivered plans under real-world conditions, considering setup, range, and motion uncertainties.

## Materials and methods

### Patient data and planning

Twenty-three lung cancer and trachea adenoid cystic carcinoma patients treated in SPHIC from March to May 2021 were analyzed retrospectively (ethical approval by the Institutional Research Board of SPHIC, SPHIC-MP-2020-04, RS). These patients were treated with protons, carbon ions, or a combination of the two modalities.

During the 4D CT scan, motion monitoring and gating were performed using the Anzai Respiratory Gating System (AZ-733V, Anzai Medical Co Ltd, Japan). The respiratory cycle was divided into 10 phases denoted by the exhale or inhale branch and the relative amplitude between the minimum and maximum positions. The system continuously monitored respiratory motion via a pressure sensor embedded in a belt worn by the patient. An average CT was reconstructed using the gating phases: exhale 20% (Ex20), end exhale (Ex0), and inhale 20% (In20). The gross tumor volume (GTV) was contoured by a physician on the average CT. The internal GTV (iGTV) was defined as the volume encompassing the GTV on all 4D CT phases. The iGTV were expended with a margin of 0.5-1.0 cm to generate the clinical target volume (CTV).^4^, ^20^

The delivered treatment plans were optimized using the SyngoRT (V13c, SIEMENS, Germany) TPS. All patients were planned using planning target volume (PTV)-based plans. A margin of 0.5-0.7 cm was implemented laterally and 0.7-1.5 cm along the beam direction on the GTV/CTV to generate the PTV (PTV-G/PTV-C). The plans were optimized on the average CT. The relative linear stopping power (RLSP) of the iGTV was overwritten to 0.95-1.05 g/cm^2^. All plans were implemented with a spot size full width half maximum that was five times the lateral raster spacing (spot spacing ≤0.47σ). The plan was optimized to provide at least 99% of the GTV/iGTV/CTV was covered by 95% of the prescribed dose (PD) (D99% > 95%), and 90% of the PTV was covered by 90% of the PD (D90% > 90%). In some cases, compromising the target coverage had been necessary to meet dose constraints for organs at risk (OARs). Among the enrolled patients, 2 received treatment with protons only, 16 received treatment with carbon ions only, and 5 patients received proton treatment followed by carbon ion sequential boost for the GTV. Simultaneously integrated boost (SIB) plans were applied for 18 patients.

A total of 29 plans were optimized for the treatment of the enrolled patients. Multifield-optimization (MFO) was applied in 24 plans, and the remaining plans were optimized with single-field optimization (SFO). Table 1 provides an overview of the patient and plan data, and Table A1 in the supplementary file includes more detailed patient and plan information. During the treatment delivery, the gating phases were identical to the planning phases (Ex20-Ex0-In20). The gating was triggered when the patient's breathing fell within the predefined gating window.

**Table 1:** Patient and plan data.

	Classify	Counts/Median	Range
No. of Pat	-	23	-
Diagnose	NSCLC	17	-
	SCLC	2	-
	TACC	4	-
No. of plans	-	29	-
Ion	C	22	-
	P	7	-
PD* GTV	-	70	60-77
PD* CTV	-	59.4	50.6-69.3
Fractions	-	22	10-26
Tech A.	SIB	22	-
	Standard	7	-
Tech B.	MFO	24	-
	SFO	5	-
No. Beams	Two	18	-
	Three	27	-
	Four	1	-
GTV (ccm)	-	50.1	1.8-223.0
CTV (ccm)	-	147.0	15.8-433.6
GTV Motion GW (mm)	-	1.8	0.5-4.2
CTV Motion GW (mm)	-	1.7	0.7-4.0

**Abbreviations:** Pat, patient; PD, prescribed dose; Tech, Technology in planning; GW, gating window; NSCLC, non-small cell lung cancer; SCLC, small cell lung cancer; TACC, Trachea Adeno carcinoma; SIB, simultaneously boost; standard, plans without using SIB; MFO, multiple field optimization; SFO, single field optimization.

* unit of PD is Gy(RBE).

### Statistics-founded robust evaluation strategy

A statistical evaluation (SE) approach was developed by Souris et al.^21^ The authors evaluated the robustness of each plan by simulating 300 treatment scenarios that accounted for setup error, range uncertainty, and breathing motion. Each scenario consisted of 35 fractions with the same systematic range and setup error, but different random setup and motion errors were included. Robustness was evaluated using a 5th and 95th dose volume histogram (DVH) band, which defined the envelope of expected dose degradation for 90% of the treatment errors. In this study, we introduced a modified approach called worst scenarios statistical evaluation (WSSE), which is based on the SE method by Souris et al.^21^ Treatment protocols are typically designed so that around 90% of patients receive a minimum dose to the CTV of at least 95% of the nominal dose, as suggested by van Herk et al.^22^ While the SE method evaluates the worst 5% of scenarios by considering the 5th percentile of target D95%/V95% values, our WSSE method evaluates the worst 10% of scenarios by considering the 10th percentile of these values. This approach effectively doubles the amount of data used for the worst-case analysis, providing a more robust representation of the minimum dose coverage that 90% of patients are likely to receive. Additionally, the WSSE method uses the 90th percentile (instead of the 95th percentile used in the SE method) of target V107% and OAR doses, effectively considering the worst 10% of OAR data instead of the worst 5%, to represent the worst-case high dose volumes and OAR doses for 90% of patients.

### Dose reconstruction in the research TPS

The proton and carbon-ion beam data from SyngoRT in SPHIC was converted into a format that could be used by TRiP4D for dose re-calculation. The details regarding the conversion of the proton and carbon-ion beam model from SyngoRT to TRiP4D and the subsequent dosimetric comparison have been discussed in a separate publication.^23^ In summary, TRiP4D was assessed for its accuracy in reproducing doses calculated by the clinically used SyngoRT system. The results showed that TRiP4D's absorbed dose calculations for proton and carbon ion beams closely matched SyngoRT's, with gamma passing rates above 99%. However, TRiP4D slightly underestimated the RBE-weighted dose by an average of −1.26%. Despite this, TRiP4D demonstrated greater efficiency in calculating the RBE-weighted dose and proved effective for evaluating doses delivered to moving targets, with the observed discrepancies deemed acceptable.

### 3D and 4D Robust evaluation

The 3D worst-case scenario statistical evaluation (3DWSSE) considers both range and setup errors. Range errors were modeled by scaling the CT Hounsfield unit (HU) to the RLSP conversion curve. Setup errors were modeled by shifting the beam isocenter. 4D worst scenarios statistical evaluation (4DWSSE) considering motion-induced dosimetric degradation on top of the 3DWSSE. The degradation caused by motion was estimated by distributing the particles in each spot equally on the treatment gating phases (Ex20-Ex0-In20). The deformable image registration metrics between Ex20 and Ex0, and between In20 and Ex0 were acquired using the software Plastimatch.^24^ 4D dose distribution was calculated by deforming scanning spots and range on the reference CT (Ex0 phase).^25^

The RLSP override of the iGTV volume was not applied in the assessment. For each plan, 100 treatment scenarios were simulated. Each treatment scenario contained the accumulated dose distribution same number of fractions as the prescribed number of fractions, or 10 fractions if the number of prescribed fractions exceeds 10.^26^ Systematic range error, systematic setup errors, and random setup errors were sampled individually from Gaussian distribution with zero mean and standard deviations (SDs) of 1.6%, 0.4 mm, and 1.7 mm, respectively. The setup error was sampled in all three spatial directions.

The worst scenarios conventional evaluation (WSCE) method evaluates the effect of range and setup uncertainties on dose distribution by calculating the worst-case scenario that could occur during treatment.^27^ In this study the conventional 3D worst scenarios conventional evaluation (3DWSCE) and 4D worst scenarios conventional evaluation (4DWSCE) with 21 different scenarios were also performed. The 3DWSCE considers static CT to evaluate the worst-case scenario based on spatial variations alone, whereas the 4DWSCE incorporates phase changes by analyzing 4D CT data. For these evaluations, the range uncertainty was set to ±3%, and the setup uncertainty in x, y, and z directions was set to ±5 mm.^28^

The dosimetric values, including ΔD95%, ΔV95%, and ΔV107% for CTV and GTV, ΔDmean for Lungs-iGTV and Heart, and ΔD1cc for trachea and esophagus, were evaluated under the 3DWSSE, 4DWSSE, 3DWSCE, and 4DWSCE methods. The relative dose deviations of these values were compared with the nominal 3D dose distribution of the reference plan.

### Statistical analysis

We performed statistical analysis using Welch's *t* test to compare the means of two independent planning strategies or targets, ie, MFO-SFO, Carbon-Proton, SIB-Standard, and CTV-GTV. Differences were considered significant if *P* < .05.

Pearson correlation coefficient was performed for the following data: a) between the GTV/CTV motion and the deviation of target (GTV/CTV) doses (ΔD95%, ΔV95%, ΔV107%), b) between the PTV-G/PTV-C doses (D95%, V95%, V107%) and the deviation of GTV/CTV doses, and c) between the target volume with 5 mm isotropic expansions (Target+5 mm) and the deviation of CTV/GTV doses. An absolute *r* value close to 1 indicates a strong correlation.

## Results

### Evaluation of target dose deviations

**Figure 1:**
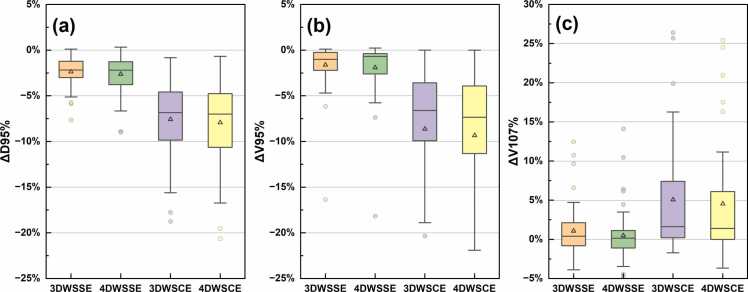
Target dose deviations: (a) ΔD95%, (b) ΔV95%, and (c) ΔV107%. The box limits represent the 25th and 75th percentiles, the center line indicates the medians, and the triangle symbol indicates the means. Whiskers are determined by extending 1.5 times the interquartile range from the 25th and 75th percentiles. Outlines are represented by dots. WSSE, worst scenarios statistical evaluation, WSCE, worst scenarios conventional evaluation.

The target dose deviations under 3DWSSE, 4DWSSE, 3DWSCE, and 4DWSCE are shown in Figure 1. The overall target dose ΔD95% was −2.37% ± 1.55% (mean ± SD) and −2.62% ± 2.08% for 3DWSSE and 4DWSSE, respectively. The target ΔV95% was −1.62% ± 2.44% and −1.91% ± 2.80% for 3DWSSE and 4DWSSE, respectively. For high dose volumes, the ΔV107% increased by 1.08% ± 3.11% and 0.49% ± 3.21% under 3DWSSE and 4DWSSE, respectively.

**Figure 2:**
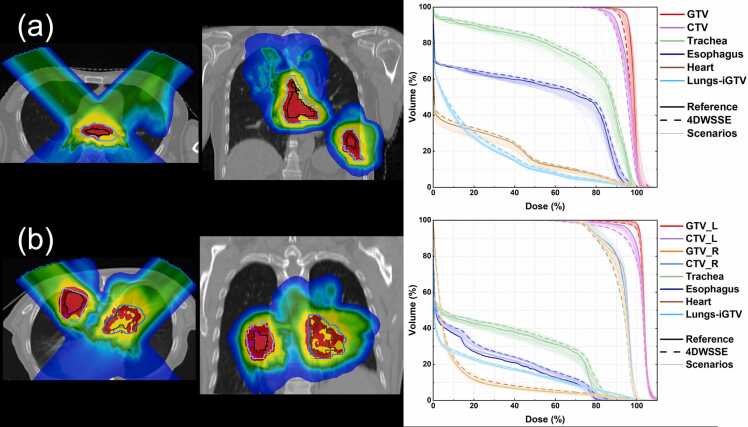
The nominal dose distribution of the reference plan and DVH for two plans, No. 20 (a) and No. 23 (b). The solid lines indicate the nominal DVH of the reference plan, and the dashed lines represent the DVH under 4DWSSE. The thinner solid line indicate the DVH of all scenarios under 4D robustness evaluation. WSSE, worst-case scenarios statistical evaluation.

The worst ΔV95% under 4DWSSE was −18.16% for the GTV of plan No.20. The dose distributions and DVH of plan No.20 are shown in Figure 2a. The two outlier points of ΔD95% under 4DWSSE were −8.91% for CTVp of plan No.16, and −8.97% for CTV-Left of plan No.23. The dose distributions and DVH of plan No.23 are shown in Figure 2b.

The overall target dose deviation ΔD95% under 3DWSCE and 4DWSCE were −7.58% ± 4.17% (mean±SD) and −7.93% ± 4.62%, respectively. The ΔV95% under 3DWSCE and 4DWSCE were −8.63% ± 7.96% and −9.34% ± 8.95%, respectively.

### Statistical analysis of target doses

Table 2 presents the mean ± SD values and the statistical analysis of target dose deviations under 3DWSSE and 4DWSSE. Significant differences (*P* < .05) were observed for the target ΔD95% between SIB GTV and SIB CTV targets, as well as for the target ΔV107% between carbon and proton beams, under both 3DWSSE and 4DWSSE. Furthermore, a significant difference was found for the target ΔV107% between SIB GTV and SIB CTV targets under 4DWSSE.

**Table 2:** Statistical analysis of the target dose deviations under different planning strategies or targets and the Pearson-correlation.

Volume /Dose	Counts	ΔD95% (relative to PD, %)		ΔV95% (relative to PD, %)		ΔV107% (relative to PD, %)	
Parameters							
		3DWSSE	4DWSSE	3DWSSE	4DWSSE	3DWSSE	4DWSSE
Welch's *t* test							
Carbon	43	−2.40 ± 1.60	−2.59 ± 2.08	−1.70 ± 2.66	−1.86 ± 2.80	0.39 ± 2.51	−0.24 ± 2.36
Proton	13	−2.26 ± 1.42	−2.71 ± 2.15	−1.36 ± 1.56	−2.06 ± 2.54	3.36 ± 3.85	2.9 ± 4.43
*P* value	-	.791	.851	.664	.818	.002*	.001*
SIBH	23	−1.70 ± 1.13	−1.55 ± 1.18	−2.02 ± 3.53	−2.11 ± 3.84	1.54 ± 2.54	1.09 ± 2.43
SIBL	23	−3.14 ± 1.67	−3.68 ± 2.21	−1.19 ± 1.05	−1.46 ± 1.20	−0.03 ± 3.06	−0.95 ± 2.83
Standard	10	−2.12 ± 1.39	−2.63 ± 2.28	−1.67 ± 1.59	−2.45 ± 2.72	2.58 ± 3.74	2.39 ± 4.35
*P* value(H-L)	-	.001*	.002*	.285	.440	.065	.012*
*P* value(H-N)	-	.371	.079	.764	.803	.357	.279
MFO	48	−2.39 ± 1.53	−2.60 ± 2.07	−1.82 ± 2.58	−2.12 ± 2.95	0.98 ± 2.95	0.41 ± 3.22
SFO	8	−2.25 ± 1.76	−2.69 ± 2.27	−0.42 ± 0.55	−0.62 ± 1.00	1.69 ± 4.09	0.97 ± 3.38
*P* value	-	.818	.917	.137	.163	.552	.653
Pearson correlation coefficient							
Motion	56	−0.160	−0.230	−0.157	−0.269	0.080	−0.002
Target+5 mm	56	0.222	0.136	0.545^a^	0.496	0.243	0.352
PTV-C/PTV-G	56	0.471	0.423	0.598^a^	0.637^a^	−0.338	−0.376

**Abbreviations:** PD, prescribed dose; SIBH, GTV target of SIB plans; SIBL, CTV target of SIB plans; Standard, plans without using SIB; MFO, multifield-optimization; SFO, single-field optimization, *P* value(H-L), *P* value (SIBH-SIBL), *P* value(H-N), *P* value(SIBH- Standard).

* *P* value < .05.

a Pearson correlation coefficient *r* > 0.5.

The Pearson-correlation between the various dosimetric parameters was generally very low, with values below 0.5. However, comparatively high correlations were observed between the PTV V95% and target (GTV/CTV) ΔV95%, with values of 0.598 and 0.637 under 3DWSSE and 4DWSSE, respectively. These results suggest that achieving higher PTV V95% coverage could generally lead to better plan robustness with respect to the target V95%.

### Evaluation of OAR dose deviation

**Figure 3:**
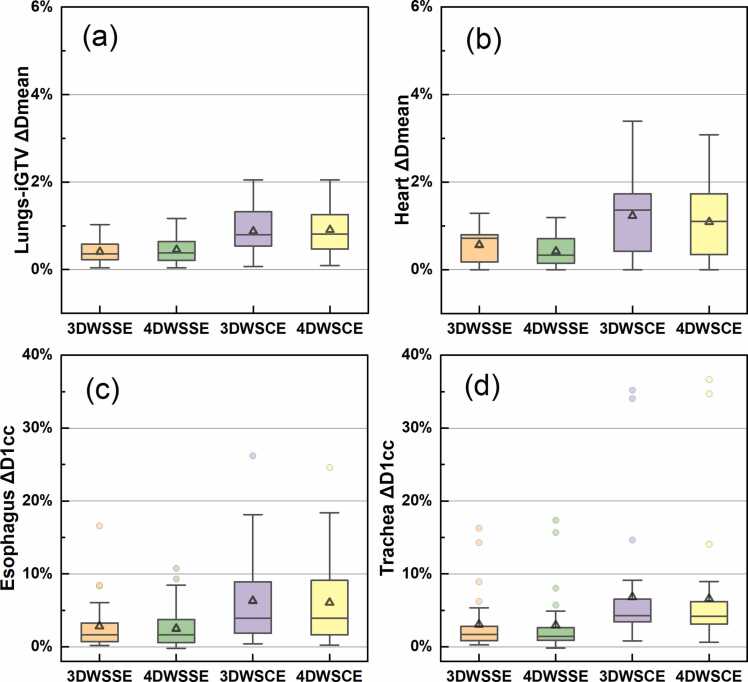
OAR dose deviations compared between WSSE/WSCE and the reference values: (a) ΔDmean of Lungs-iGTV, (b) ΔDmean of heart, (c) D1cc of esophagus, and (d) D1cc of trachea. The box limits represent the 25th and 75th percentiles, the center line indicates the median, and the triangle symbol indicates the mean. Whiskers are determined by extending 1.5 times the interquartile range from the 25th and 75th percentiles. Outlines are represented by dots. WSSE, worst scenarios statistical evaluation, WSCE, worst scenarios conventional evaluation.

The OAR dose deviations under 3DWSSE, 4DWSSE, 3DWSCE, and 4DWSCE are shown in Figure 3. The ΔDmean for Lungs-iGTV and Heart were generally low, with all deviations ≤1.19% of the PD for WSSE and ≤2.31% of the PD for WSCE.

**Figure 4:**
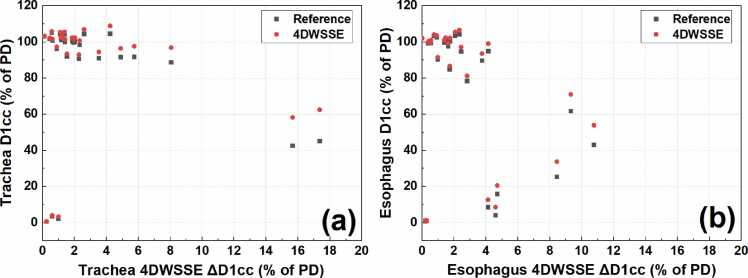
ΔD1cc under 4DWSSE vs D1cc in the reference plan and the absolute D1cc under 4DWSSE for esophagus (a) and trachea (b). PD, prescribed dose, WSSE, worst scenarios statistical evaluation.

The ΔD1cc for esophagus and trachea was 2.52% ± 2.83% (mean ± SD) and 2.97% ± 4.18% under 4DWSSE, respectively. Figure 4 illustrates the correlation between ΔD1cc under 4DWSSE and the nominal D1cc in the reference plan, as well as the absolute D1cc under 4DWSSE for the esophagus and trachea. Plans with ΔD1cc values greater than or equal to 5% typically had low corresponding nominal D1cc values, and the D1cc values under 4DWSSE never exceeded 100% of the PD, so any overdosing in these cases can be disregarded.

### Statistical analysis of OAR dose indices

The statistical analysis of the OAR dose deviations, together with mean ± SD values under 3DWSSE and 4DWSSE, are presented in Table A2 in the supplementary file. Similar to the target dose V107%, the proton plans exhibit relatively higher deviations than the carbon plans for the OAR doses. There were no significant differences observed among the different plan strategies.

## Discussion

In this study, we evaluated the robustness of clinical proton and carbon-ion plans for lung and trachea cancer patients. We presented the deviation of the target dose and OAR doses under 3D and 4D statistical-based worst-case scenario robust evaluations and compared different planning strategies.

This study differs from previous comparisons between PTV-based plans and 3D or 4D robust optimization plans that mainly focused on the robust optimization techniques. Instead, this study retrospectively analyzed clinical treatment plans, providing more clinically relevant data for reference. These treatment plans employed a series of measures to reduce the uncertainties arising from setup error, range uncertainty, and motion, which are the main sources of intra-fractional uncertainties in particle planning. Moreover, a comprehensive statistical evaluation WSSE approach was used, and it takes into account the effects of fractionation.^21^, ^29^ The WSSE approach provided a conservative estimate of the worst possible doses for the target area and OARs in 90% of the patients. Comparing plans against previous clinical experience enables the production of comparative data for future treatment plans incorporating robust optimization, ensuring a consistent clinical output.

Park et al^26^ used a statistical simulation approach to retrospectively evaluated the robustness of the clinically approved proton plans of 20 patients with locally advanced NSCLC, 10 patients with prostate cancer, and 1 brain cancer. Passively scattered beam treatment planning was used for all lung cancer patients. They performed 600 dose recalculations (60 scenarios multiplied by 10 fraction) for each plan and averaged all resulting dose distributions to quantify the deviation between the expected and nominal DVH values. It was found that the deviation of ITV V74Gy (100%) for lung cancer patient was reduced by −1.1% ± 1.5%. Our study demonstrates that, under 3DWSSE, there was a comparable average reduction in the target dose, with ΔV95% −1.62% ± 2.44%, but with greater variation for certain patients. It is noteworthy that our plans were based on pencil beam scanning beams, in contrast to the passive scattering beams utilized in other literature.

Based on the statistical analysis, there was a significant difference (*P* value = .002) between the ΔD95% for CTV (−3.68%) and GTV (−1.55%) under 4DWSSE. These results suggest that, for SIB plans, CTV targets are more sensitive to uncertainties compared to GTV targets in terms of the volume receiving a specific dose percentage. In fact, both of the two ΔD95% outliers in Figure 1 were CTV targets. Specifically, we observed a reduction of −8.97% and −8.91% under 4DWSSE for plans No. 23 (CTV-L) and plan No. 16 (CTV), respectively. One reason is that the dose gradient may be steeper for the CTV compared to the GTV, which means that small setup or range error can leading to a lower dose in the CTV (CTV D95%) compared to the GTV (GTV D95%). Another reason is that the CTV includes more low-density lung tissue, which makes CTV more sensitive to range errors compared to the GTV.

The largest reduction in ΔV95% was observed for GTV in plan No. 20, which decreased by −18.16% under 4DWSSE. Figure 2a shows that the trachea was attached to the GTV, and to avoid invalidating 95% PD into the trachea, the GTV coverage V95% was compromised. Due to the high dose gradient around the surrounding area, even a small setup error could cause a significant reduction in the volume of GTV receiving 95% PD.

Badiu et al^30^ conducted a study on robust optimization in proton therapy for thirteen lung cancer patients, using both CE via Raystation and SE via the Monte Carlo engine MCsquare.^31^ Although they did not report the deviation of target doses under robust evaluation, based on the reported data, it can be estimated that the worst ΔD98% would be less than 1% under SE. Graeff et al^14^ evaluated the robustness of 4D-optimized carbon-ion plans for four lung cancer patients, and the results showed that for all patients, the target V95% was always higher than 95%, indicating that a ΔV95% of not less than −5% was achieved for all patients. Despite the numerous efforts to mitigate dose uncertainties, the PTV-based plans still have lower D95% or V95% coverage compared to reported robustly or 4D-optimized particle plans. Although none of the evaluated plans had both ΔV95% and ΔD95% exceeding 5%, it is still advisable to use a robust or 4D-optimized plan if available. A proper, robust evaluation procedure should be performed for particle therapy.

The 3DWSSE model considers only setup error and range uncertainty, while the 4DWSSE model takes into account motion-induced dose uncertainties in addition to these factors. The mean difference between 4DWSSE and 3DWSSE was found to be −0.25% ± 0.82% and −0.29% ± 0.77% for ΔD95% and ΔV95%, respectively. Therefore, the use of gating to reduce motion amplitude results in relatively small motion-induced dose uncertainties compared to those caused by setup error and range uncertainty.

The uncertainty models used in this study were based on the methodology outlined by Souris et al, which included systematic and random setup errors (σ = 0.4 and 1.7 mm), as well as systematic range uncertainties (σ = 1.6%). However, as IGRT systems, immobilization practices, and therapist experience can vary across institutions, the uncertainty model employed in this study may not fully capture all motion and setup uncertainties. To enhance the accuracy of robustness evaluations, we recommend the continued refinement of uncertainty models that account for these variations.

We observed approximately 5% lower in the WSCE compared to the WSSE method, for example the 4DWSCE showing a target ΔD95% of −7.93% ± 4.62% and the 4DWSSE showing a target ΔD95% of −2.62% ± 2.08%. The WSCE approach providing a more conservative evaluation of dose deviations; however, it might underestimate target dose coverage. On the other hand, WSSE evaluates dose deviations using a broader statistical approach, which captures a wider range of uncertainties, but may not fully account for rare, extreme worst-case errors that potentially underestimating the risks of large setup or range errors. These findings highlight the importance of long-term clinical validation to assess the ability of both methods to predict real-world outcomes, and to determine the optimal strategy for ensuring robust and reliable treatment planning.

This study has some limitations. First, the interplay effect was not considered in the 4D dose evaluation, and additional evaluations are needed to verify its impact. Second, the effect of intra- and inter-fractional variation of breathing motion and anatomical changes was not evaluated, which may also influence the dose received by the patient. Also, a perfect correlation between the breathing signal and the internal motion was assumed. Third, due to the limited number of cases in this study, a potential bias may occur in the OAR evaluation because of the different locations of the target and OARs, as well as the fact that the PD for carbon-ion therapy is lower than for proton therapy. A more comprehensive study with a larger patient cohort is recommended to provide more robust statistical data for OAR dose evaluations. Fourth, since TRiP4D employs an analytical dose algorithm, dose calculation accuracy may be affected by heterogeneous tissues in thoracic patients. Therefore, Monte Carlo-based calculations are recommended when available.

## Conclusion

The impact of setup error, range uncertainty, and motion on dose distributions was evaluated based on 29 clinically treated proton and carbon-ion beam plans. A comprehensive robustness evaluation method was implemented. Our results demonstrate that the target coverage deviation is acceptable for most patients. The induced uncertainties had limited impact on the mean doses for Lungs-iGTV and heart. While for some plans with relatively low absolute esophagus and trachea D1cc values, ΔD1cc > 5% of PD was observed, this does not pose a significant clinical concern since the absolute esophagus and trachea D1cc values were still within an acceptable range. Despite applying various strategies to mitigate uncertainties in the clinical particle plans, some plans still have low ΔD95% or ΔV95% values. Typically, low values of ΔD95% are observed for CTV targets, while low ΔV95% values are observed for GTV targets. Conventional robustness evaluations often lead to significantly reduced target coverage by incorporating highly unlikely worst-case scenarios. In contrast, the comprehensive WSSE method effectively identifies critical patients without overestimating and overcompensating for unrealistic error scenarios.

## Ethics statement

The study was approved by the Institutional Research Board of Shanghai Proton and Heavy Ion Center in accordance with the ethical principles set out in the Declaration of Helsinki (approval number SPHIC-MP-2020-04, RS). All participants provided informed consent prior to their inclusion in the study. The authors confirm that informed consent was obtained from all individual participants included in the study.

## Funding

This project is funded by the International Postdoctoral Exchange Fellowship Program (CN 2020016) and Shanghai Pujiang Program (23PJ1411100). The publication is funded by the Open Access Publishing Fund of GSI Helmholtzzentrum fuer Schwerionenforschung.

## CRediT authorship contribution statement

YS: Yinxiangzi Sheng. LV: Lennart Volz. JM: Jingfang Mao. JC: Jian Chen. TS: Timo Steinsberger. WW: Weiwei Wang. JS: Jiayao Sun. RH: RongCheng Han. MD: Marco Durante. CG: Christian Graeff. Contributions:. Conceptualization: CG, MD. Data curation: YS, JM, RH, JS. Formal Analysis: YS, LV. Funding acquisition: YS, CG. Investigation: YS, LV, CG. Methodology: YS, LV, CG. Project administration: MD**.** Resources: JC, JM. Software: YS, LV. Supervision: CG, MD. Validation: YS. Visualization: YS, LV. Writing – original draft: YS. Writing – review and editing: all authors.

## Declaration of Conflicts of Interest

The authors declare that they have no known competing financial interests or personal relationships that could have appeared to influence the work reported in this paper.

## Declaration of Generative AI and AI-Assisted Technologies in the Writing Process

During the preparation of this work, the authors used OpenAI's GPT language model via OpenAI API in order to improve readability and language. After using this tool, the authors reviewed and edited the content as needed and take full responsibility for the content of the publication.
