# Supplementary material for: Comprehensive Robustness Evaluation of Proton and Carbon-Ion Plans in Thoracic Cancer Treatment
Source: Int J Part Ther. 2025 Jun 24;17:101195. doi: 10.1016/j.ijpt.2025.101195 (PMC12318338; doi:10.1016/j.ijpt.2025.101195)
Supplement: Supplementary file 1 — Supplementary material [file mmc1.docx]

**Table A.1. Detailed patient and plan information**

| **Patient No.** | **Diagnosis** | **Plan No.** | **Ion** | **PD GTV**† | **PD CTV**† | **Fx** | **Tech A.** | **Tech B.** | **No. Beams** | **GTV (ccm)** | **CTV  (ccm)** | **GTV Motion GW (mm)** | **CTV Motion GW (mm)** |
| --- | --- | --- | --- | --- | --- | --- | --- | --- | --- | --- | --- | --- | --- |
| Pat1 | NSCLC | Plan1 | C | 70 | 63 | 20 | SIB | MFO | 2 | 170.2 | 37.5 | 0.8 | 0.7 |
| Pat2 | Trachea ACC | Plan2 | P | - | 50.6 | 23 | Standard | SFO | 2 | - | 63.4 | 1.3 | 1.3 |
| Pat3 | NSCLC | Plan3 | P | - | 50.6 | 23 | Standard | MFO | 3 | 13.4 | 125.1 | 1.8 | 2.0 |
|  |  | Plan4 | C | 11.4 | - | 3 | Standard | SFO | 2 |  |  |  |  |
| Pat4 | NSCLC | Plan5 | P | - | 50.6 | 23 | Standard | MFO | 2 | 3.3 | 130.5 | 3.2 | 3.6 |
|  |  | Plan6 | C | 11.4 | - | 3 | Standard | MFO | 2 |  |  |  |  |
| Pat5 | NSCLC | Plan7 | C | 75 | 67.5 | 20 | SIB | SFO | 2 | 23.5 | 114.6 | 3.7 | 3.8 |
| Pat6 | NSCLC | Plan8 | C | 77 | 69.3 | 22 | SIB | MFO | 3 | 214.2 | 410.9 | 1.3 | 1.7 |
| Pat7 | NSCLC | Plan9 | C | 73.5 | 66.15 | 21 | SIB | MFO | 2 | 199.9 | 433.6 | 0.8 | 0.8 |
| Pat8 | NSCLC | Plan10 | P | 44 | 39.6 | 20 | SIB | MFO | 3 | 82.2 | 377.9 | 1.9 | 1.6 |
|  |  | Plan11 | C | 19 | 17.1 | 5 | SIB | MFO | 3 |  |  |  |  |
| Pat9 | SCLC | Plan12 | P | 44 | 39.6 | 20 | SIB | MFO | 2 | 39.1 | 150.0 | 1.3 | 1.4 |
|  |  | Plan13 | C | 19 | 17.1 | 5 | SIB | MFO | 2 |  |  |  |  |
| Pat10 | SCLC | Plan14 | P | 44 | 39.6 | 20 | SIB | MFO | 3 | 40.2 | 143.4 | 2.0 | 1.8 |
|  |  | Plan15 | C | 19 | 17.1 | 5 | SIB | MFO | 3 |  |  |  |  |
| Pat11 | NSCLC | Plan16 | C | 70 | 63 | 10 | SIB | MFO | 2 | 10.2 | 46.2 | 3.4 | 3.4 |
| Pat12 | Trachea ACC | Plan17 | C | 66 | 59.4 | 20 | SIB | MFO | 2 | 10.2 | 23.7 | 0.9 | 0.9 |
| Pat13 | NSCLC | Plan18 | P | - | 50.6 | 23 | Standard | MFO | 2 | - | 135.4 | 4.2 | 4.0 |
| Pat14 | NSCLC | Plan19 | C | 70 | - | 20 | Standard | MFO | 2 | 85.5 | - | 1.8 | - |
| Pat15 | NSCLC | Plan20 | C | 77 | 69.3 | 22 | SIB | MFO | 4 | 52.2 | 265.8 | 2.4 | 2.0 |
| Pat16 | NSCLC | Plan21 | C | 77 | 69.3 | 22 | SIB | MFO | 3 | 223.0 | 416.8 | 1.1 | 1.2 |
| Pat17 | Trachea ACC | Plan22 | C | 66 | 59.4 | 22 | SIB | MFO | 2 | 151.9 | 179.8 | 1.1 | 1.1 |
| Pat18* | NSCLC | Plan23 | C | 64/  60* | 57.6/  54* | 16 | SIB | MFO | 3 | 47.9/  69.8* | 114.6/  188.7* | 2.5/  3.1* | 2.4/  3.3* |
| Pat19 | NSCLC | Plan24 | C | 77 | 69.3 | 22 | SIB | MFO | 2 | 72.2 | 172.9 | 0.5 | 0.8 |
| Pat20 | NSCLC | Plan25 | C | 77 | 69.3 | 22 | SIB | MFO | 3 | 40.5 | 225.9 | 2.7 | 2.6 |
| Pat21 | NSCLC | Plan26 | C | 77 | 69.3 | 22 | SIB | MFO | 2 | 86.0 | 235.4 | 2.6 | 2.6 |
| Pat22# | NSCLC | Plan27# | C | 60 | 54 | 10 | SIB | SFO | 2 | 1.8 | 15.8 | 1.6 | 1.6 |
|  |  | Plan28# | C | 60 | 54 | 10 | SIB | SFO | 2 |  |  |  |  |
| Pat23 | Trachea ACC | Plan29 | C | 72.6 | 66 | 22 | SIB | MFO | 3 | 27.1 | 147.0 | 2.3 | 2.5 |

*Abbreviations*: PD=prescribed dose, Fx=prescribed fractions, Tech.=Technology in planning, GW=gating window, NSCLC=Non-small cell lung cancer, SCLC= small cell lung cancer, Trachea-ACC= Trachea Adeno carcinoma, SIB=simultaneously boost plans, Standard=plans without using SIB, MFO=Multiple field optimization, SFO=Single field optimization

† unit of PD is Gy(RBE)

* This plan have four targets, GTV-L (64Gy(RBE)), GTV-R(60 Gy(RBE)), CTV-L(57.6 Gy(RBE)), and CTV-R(54 Gy(RBE))

# Two plans used the same parameters except for beam angle, each plan treated 5 fractions and alternatively everyday.

**Table A.2. statistical analysis of the OAR dose deviations under different planning strategies or targets and the Pearson-correlations**

| **Volume /**  **Dose**  **Parameters** | | **Lungs-iGTV**  **ΔDmean** | | **Heart**  **ΔDmean** | | **Esophagus**  **ΔD1cc** | | **Trachea**  **ΔD1cc** | |
| --- | --- | --- | --- | --- | --- | --- | --- | --- | --- |
|  |  | **3DWSSE** | **4DWSSE** | **3DWSSE** | **4DWSSE** | **3DWSSE** | **4DWSSE** | **3DWSSE** | **4DWSSE** |
| **Carbon** | 22 | 0.43±0.27 | 0.51±0.31 | 0.55±0.47 | 0.41±0.38 | 2.74±3.66 | 2.48±2.86 | 2.74±3.61 | 2.52±3.77 |
| **Proton** | 7 | 0.38±0.15 | 0.29±0.14 | 0.63±0.24 | 0.46±0.24 | 3.16±2.98 | 2.62±2.94 | 4.16±4.86 | 4.39±5.34 |
| **P-value** | - | 0.645 | 0.112 | 0.635 | 0.761 | 0.782 | 0.913 | 0.412 | 0.310 |
| **SIB** | 22 | 0.44±0.24 | 0.51±0.32 | 0.56±0.45 | 0.42±0.38 | 2.27±2.11 | 2.17±2.22 | 3.33±4.35 | 3.24±4.66 |
| **Standard** | 7 | 0.33±0.23 | 0.29±0.25 | 0.59±0.33 | 0.41±0.28 | 4.65±5.94 | 3.59±4.29 | 2.30±1.94 | 2.15±2.11 |
| **P-value** | - | 0.279 | 0.113 | 0.870 | 0.954 | 0.114 | 0.257 | 0.553 | 0.558 |
| **MFO** | 48 | 0.44±0.25 | 0.5±0.33 | 0.61±0.42 | 0.45±0.35 | 2.69±3.32 | 2.28±2.33 | 3.38±4.10 | 3.29±4.43 |
| **SFO** | 8 | 0.29±0.16 | 0.29±0.23 | 0.34±0.38 | 0.25±0.33 | 3.56±4.44 | 3.65±4.80 | 1.64±2.58 | 1.46±2.44 |
| **P-value** | - | 0.217 | 0.191 | 0.201 | 0.253 | 0.617 | 0.333 | 0.373 | 0.384 |

*Abbreviations: PD-prescribed dose, SIBH-GTV target of SIB plans, SIBL-CTV target of SIB plans, Standard-plans without using SIB, MFO- multifield-optimization, SFO - single-field optimization*
